# Techno‐Economic Analysis of Membrane‐Based Purification Platforms for AAV Vector Production

**DOI:** 10.1002/bit.29034

**Published:** 2025-05-29

**Authors:** Juan J. Romero, Eleanor W. Jenkins, Jacob I. Monroe, S. Ranil Wickramasinghe, Xianghong Qian, Dibakar Bhattacharyya, Scott M. Husson

**Affiliations:** ^1^ Department of Chemical and Biomolecular Engineering Clemson University Clemson South Carolina USA; ^2^ School of Mathematical and Statistical Sciences Clemson University Clemson South Carolina USA; ^3^ Ralph E. Martin Department of Chemical Engineering University of Arkansas Fayetteville Arkansas USA; ^4^ Department of Biomedical Engineering University of Arkansas Fayetteville Arkansas USA; ^5^ Department of Chemical and Materials Engineering University of Kentucky Lexington Kentucky USA

**Keywords:** bioprocess cost models, membrane design, model‐based simulation, vector‐based gene therapy

## Abstract

Technologies for large‐scale manufacturing of viral vectors for gene therapies, such as tangential flow filtration and membrane chromatography, are under development. In these early stages of process development, techno‐economic analyses are useful for identifying membrane properties yielding the greatest impact on process performance. In this study, we adapted a techno‐economic framework used for monoclonal antibody capture for adeno‐associated viral vector purification. We added mechanistic models to simulate flux decline during harvesting and separating full and empty capsids during polishing. Graphical user interfaces were added to help users explore the design search space. We selected a base process and manipulated selected variables to see their impact on large‐scale manufacturing performance. These sensitivity analyses revealed that, under the selected process conditions, increasing module capacity reduces cost of goods more effectively than increasing operational flux in tangential flow membrane filtration modules for virus harvesting. Membrane chromatography columns with relatively low dynamic binding capacity (DBC) and short residence time (RT) offered similar or better economic performance than those with high DBC and long RT. Additionally, the difference in equilibrium solid‐phase concentration between full and empty capsids as a function of salt concentration significantly affects purity.

## 1 Introduction

Gene therapy treats or prevents diseases by introducing a missing gene (addition), inactivating the expression of a defective gene (silencing), or replacing a faulty gene with a functional copy (editing) (Li et al. 2023). Gene therapy offers potential cures for conditions that otherwise require continuous medication (Ma et al. 2020). Currently, over 30 gene therapies are approved worldwide, with more than 3700 others in clinical and preclinical trials (Chancellor et al. 2023; Wang et al. 2024). In 2023 alone, the FDA approved five new gene therapy products for treating hemophilia A, sickle cell disease, Duchenne's muscular dystrophy and dystrophic epidermolysis bullosa (Senior 2024).

To exert its therapeutic effect, the genetic material must reach the nucleus of the target cells. To achieve this, one can use the natural ability of viruses to deliver genetic material into cells for self‐replication, using them as vectors (Kohn et al. 2023). Among the available vectors, adeno‐associated viral (AAV) vectors are the most used, accounting for approximately a quarter of all viral‐based gene therapies (Chancellor et al. 2023; Wang et al. 2024). AAV was first identified as a contaminant in adenovirus samples, a virus known to cause cold‐like symptoms in humans (Shieh 2022). There are at least 12 serotypes of AAV found in nature, infecting human populations at percentages ranging from approximately 15% to more than 90%. Infections are asymptomatic, and AAV replication only occurs in the presence of helper factors resulting from coinfections with other viruses such as herpesviruses, adenoviruses, and papillomaviruses (Meier et al. 2020). Nonetheless, recombinant AAV (rAAV) for therapeutic purposes can be produced using AAV‐helper‐free cell systems (Crosson et al. 2018). AAV vectors are favored for in vivo gene therapy because of their high transduction efficiency, relatively low immunogenicity, and non‐pathogenicity, among other advantages (Li et al. 2023).

Currently, there is no consensus on a standard AAV production platform, but literature and supplier information suggest a base downstream processing (DSP) platform (Lyle et al. 2023; Rieser et al. 2021; Yang et al. 2022). This includes harvesting by depth filtration using a 0.2 μm pore size membrane, concentration and diafiltration using a membrane with a 300 kDa nominal molecular weight cutoff (NMWCO), capture purification using affinity resin chromatography, and polishing purification using anion‐exchange (AEX) resin chromatography (Cytiva LifeSciences 2024; Sartorius Stedim Biotech GmbH 2017). The polishing step involves binding AAV capsids and eluting sequentially to separate full and empty capsids. The current purification technology often creates bottlenecks in the manufacturing process (Kilgore et al. 2023).

To improve production capacity and product quality, we propose an alternative process using tangential flow filtration (TFF) for harvesting, affinity membrane chromatography for capture, and AEX membrane chromatography for polishing. The reduced fouling compared to depth filtration achieved with TFF is expected to decrease flow resistance and increase throughput (Zhang et al. 2021b). Mendes et al. reported higher capacities and recovery yield for AAV harvesting using tangential flow depth filtration (TFDF, Repligen), a variation of TFF (Mendes et al. 2022). However, due to the large pore size of the membranes used in TFDF, a secondary clarification step is needed before chromatography. This issue is avoided with TFF, which does not require further clarification (van Reis and Zydney 2007).

In chromatography operations, membrane chromatography columns have the potential to increase throughput compared to traditional resin columns (Boi 2007). Peixoto et al. demonstrated the feasibility of using membranes for AAV capture and polishing (Peixoto et al. 2008).

In this study, we incorporated mechanistic models to simulate these operations into a previously developed framework that uses SuperPro Designer for flowsheet simulations (Romero et al. 2022). The objective of this framework is to help manufacturers and suppliers quickly evaluate the techno‐economic feasibility of their products and processes. Accordingly, we developed a graphical user interface for conducting sensitivity analyses. We used this tool to explore the influence of membrane parameters on process performance for a base process. The findings uncovered parameters that could lead to substantial process improvements and guide new product development.

The framework developed here distinguishes itself from other frameworks that combine mechanistic and flowsheet simulations by offering straightforward usability and reduced complexity. Unlike frameworks requiring intricate configurations across multiple tools, such as Brunet et al.'s integration of MATLAB, GAMS, and SuperPro Designer (Brunet et al. 2012), or Taras and Woinaroschy's interactive decision‐making interface (Taras and Woinaroschy 2012), this tool simplifies the process with an intuitive MATLAB‐based graphical user interface (GUI). Designed specifically for AAV process development, the GUI automates sensitivity analyses and optimizations, making modeling and optimization accessible to a wider range of users.

## 2 Methods

### 2.1 Harvesting Model

For harvesting, we implemented a resistance‐in‐series model for incompressible particles. This model uses Darcy's law (Equation 1) to describe flux decline. The permeate flux (*Q*_*p*_/*A*) is proportional to the transmembrane pressure (Δ*P*) and inversely proportional to viscosity (*μ*) and the sum of membrane and cake resistances (*R_m_* +*R_c_*).

(1) $$\frac{Q_{p}}{A}=\frac{\Delta P}{\mu \left(R_{m}+R_{c}\right)}$$

We assume that no small particles get trapped inside the membrane, causing *R_m_* to be constant parameter throughout the filtration process. Meanwhile *R_c_* increases as the cake layer forms on the membrane surface. *R_c_* is described using the constitutive relationship in Equation 2 and depends on the cake mass per unit area (*m*/*A*) and Δ*P*, with *α*_0_ and *n* as model parameters. Here, *α*₀ is a fitting constant dependent on particle size and shape, while *n* represents the compressibility index of the cake.

(2) $$R_{c}=\frac{m}{A}\alpha_{0}\Delta P^{n}$$

The cake formation rate, given by Equation 3, is proportional to the mass of solid material rejected by the membrane, represented by the permeate flow rate (*Q_p_*) multiplied by the concentration of solid particles (*C*) in the solution. It assumes 100% rejection of these particles.

(3) $$\frac{\mathrm{dm}}{\mathrm{dt}}=CQ_{p}-\mathrm{mK}Q_{f}$$

In TFF, the crossflow reduces the cake layer formation rate, and the rate is expected to be a function of shear rate. This is accounted for in the second term on the right‐hand side of Equation 3, which depends on cake mass, feed flow rate (*Q_f_*) and a constant known as the attrition factor (*K*). For a given wall shear rate (*γ*), *K* is modeled as a linear function of permeate flux (Equation 4).

(4) $$K=K_{m}Q_{p}+K_{i}$$

The material balances in Equations 5 and 6 describe the concentration and liquid volume (*V*) in the system, with *C*_0_ and *V*_0_ being the initial concentration and volume.

(5) $$C=\frac{C_{0}\cdot V_{0}-m}{V}$$

(6) $$\frac{\mathrm{dV}}{\mathrm{dt}}=-Q_{p}.$$

We used the model parameters reported by Zhang et al. (Zhang et al. 2021a) for the BioOptimal MF‐SL hollow fiber microfilter, using a yeast suspension as the ultrafiltration feed. In this model, the yeast cells form an incompressible cake. However, the cell debris in the AAV lysate might behave differently, requiring the adjustment of the compressibility index (*n*) in Equation 2. For this type of module, *Q_f_* is related to *γ* according to Equation 7, where *r* represents the fiber inner radius.

(7) $$Q_{f}=\frac{\pi r^{3}\gamma }{4}$$

### 2.2 Capture Model

We selected an instantaneous capture model with a Langmuir isotherm to describe the adsorption equilibrium. This model is suitable for membrane adsorption, a process controlled by advection rather than diffusion (Boi 2007). We adopted a modeling approach similar to Dimartino et al. (Dimartino et al. 2011), with Equation 8 as the governing equation and Equations (9), (10) describing the Danckwerts boundary conditions.

(8) $$\omega \frac{\partial C}{\partial t}+\left(1-\omega \right)\frac{\partial q}{\partial t}=-\nabla \cdot \;\left(uC\right)+\nabla \cdot \;\left(D\nabla C\right)$$

(9) $$-\left(D\nabla C\right)\cdot n=u\left(C_{\mathrm{in}}-C\right)\;\;\mathrm{at}\;x=0$$

(10) ∇C·n=0  atx=L

*ω* is porosity, u is the average flow velocity, and *q* is the product concentration in the solid phase. Dispersity (*D*) is related to flow velocity by Equation 11, with α being the dispersion coefficient. The adsorption rate is described by Equation 12, which assumes that concentration equilibrium is reached instantaneously following the Langmuir isotherm.

(11) D=α·u

(12) $$\frac{\partial q}{\partial t}=\frac{\partial q}{\partial C}\frac{\partial C}{\partial t}=\frac{K_{l}q_{\max}}{{\left(1+K_{l}C\right)}^{2}}\frac{\partial C}{\partial t}$$

The product concentration entering the membrane (*C_in_*) is modeled assuming the void volume in the housing module is perfectly mixed. Consequently, this volume (*V_mix_*) can be modeled as a continuous stirred‐tank reactor in Equation 13, with *C_feed_* as the concentration of the stream entering the module.

(13) $$\frac{\partial C_{\mathrm{in}}}{\partial t}=\frac{F}{V_{\mathrm{mix}}}\cdot \left(C_{\mathrm{feed}}-C_{\mathrm{in}}\right)$$

The model parameters (*K_l_*, *q_max_* and *α*) were fitted to breakthrough data reported by McNally et al. for AAV‐MutC particles capture using a Sartobind Phenyl‐ligand membrane (Sartorius) (McNally et al. 2020). The simulated breakthrough curve (Figure S1) agreed well with the experimental data, showing that the model adequately represents the system. The simulated DBC at 10% breakthrough was 1.07 × 10¹³ VP/mL, consistent with the experimentally reported value (1.07 × 10^13^ VP/mL). Although this model was applied to a hydrophobic interaction membrane, it can be adapted for ion exchange, affinity or multimodal membranes by changing the isotherm model and consistently adjusting Equation 12.

### 2.3 Polishing Model

For AAV, polishing step chromatography purification runs in bind‐and‐elute mode to reduce the impurity content, especially empty capsids. We must independently model the chromatography operation for empty and full capsids to simulate this process. The binding step is described using the same adsorption model applied during the capture stage (Equations (8), (9), (10), (11), (12), (13)). This assumes that all capsids bind to the membrane at the same rate and that the separation of full and empty capsids occurs during the elution phase (Di et al. 2024; Gomis‐Fons et al. 2024; Kurth et al. 2024). As a membrane model, we selected the Sartobind Q anion exchange (AEX) membrane (Sartorius). Due to the similar support matrix, we applied the same *α* and *K_l_* parameters as for the phenyl membrane used in capture, adjusting *q_max_* based on the DBC of 1.9 × 10¹³ VP/mL reported by Fan et al. for AAV serotype 2 (Fan et al. 2022).

We implemented a rate‐based desorption model (Equation 14) with species g being either full or empty capsids. Here, the decrease in solid phase concentration is proportional to the difference between the current concentration and an equilibrium concentration (*q*_*g*,*eq*_) that depends on the mobile phase salt concentration (*C_s_*). To account for the minimum conductivity required for elution to start, we included a Heaviside function that inhibits desorption (*h* = 0) when *C_s_* is below a critical value (*C_s,crit,g_*).

(14) $$\frac{\partial q_{g}}{\partial t}=-K_{e,g}\cdot \left(q_{g}-q_{g,\mathrm{eq}}\left(C_{s}\right)\right)*h\left(C_{s}-C_{s,\mathrm{crit},g}\right)$$

For simplicity *q_g,eq_* is modeled using a constitutive relationship that assumes the initial concentration (*q*_*g*,0_) decreases linearly with increasing *C_s_* until it reaches zero (Equation 15). This model requires estimating two fitting parameters: *m_q,g,eq_* and *i_q,g,eq_*, which do not have any direct physical interpretation. Alternatively, more complex models can be employed to describe capsid adsorption, such as steric mass action (Vicente et al. 2011) or multi‐component Langmuir (Gomis‐Fons et al. 2024).

(15) $$q_{g,\mathrm{eq}}\left(C_{s}\right)=\left\{\begin{array}{l} q_{g,0}\left(i_{q,g,\mathrm{eq}}-C_{s}\cdot m_{q,g,\mathrm{eq}}\right),\text{for}\;C_{s}<\frac{i_{q,g,\mathrm{eq}}}{m_{q,g,\mathrm{eq}}}\\ 0,\text{for}\;C_{s}>\frac{i_{q,g,\mathrm{eq}}}{m_{q,g,\mathrm{eq}}} \end{array}\right.$$

We modeled the salt concentration in the liquid phase using the governing equation (Equation 16) along with Danckwerts boundary conditions (Equations (9), (10)) and the inlet concentration described in Equation 13.

(16) $$\omega \frac{\partial Cs}{\partial t}=-\frac{\partial \mathrm{uC}s}{\partial x}+\frac{\partial }{\partial x}\left(D\frac{\partial Cs}{\partial x}\right)$$

Common ways to increase the conductivity include linear salt gradients or step changes. In this study, we used a two‐step increase in *C_s_*.

### 2.4 Simulation Framework

**Figure 1:**
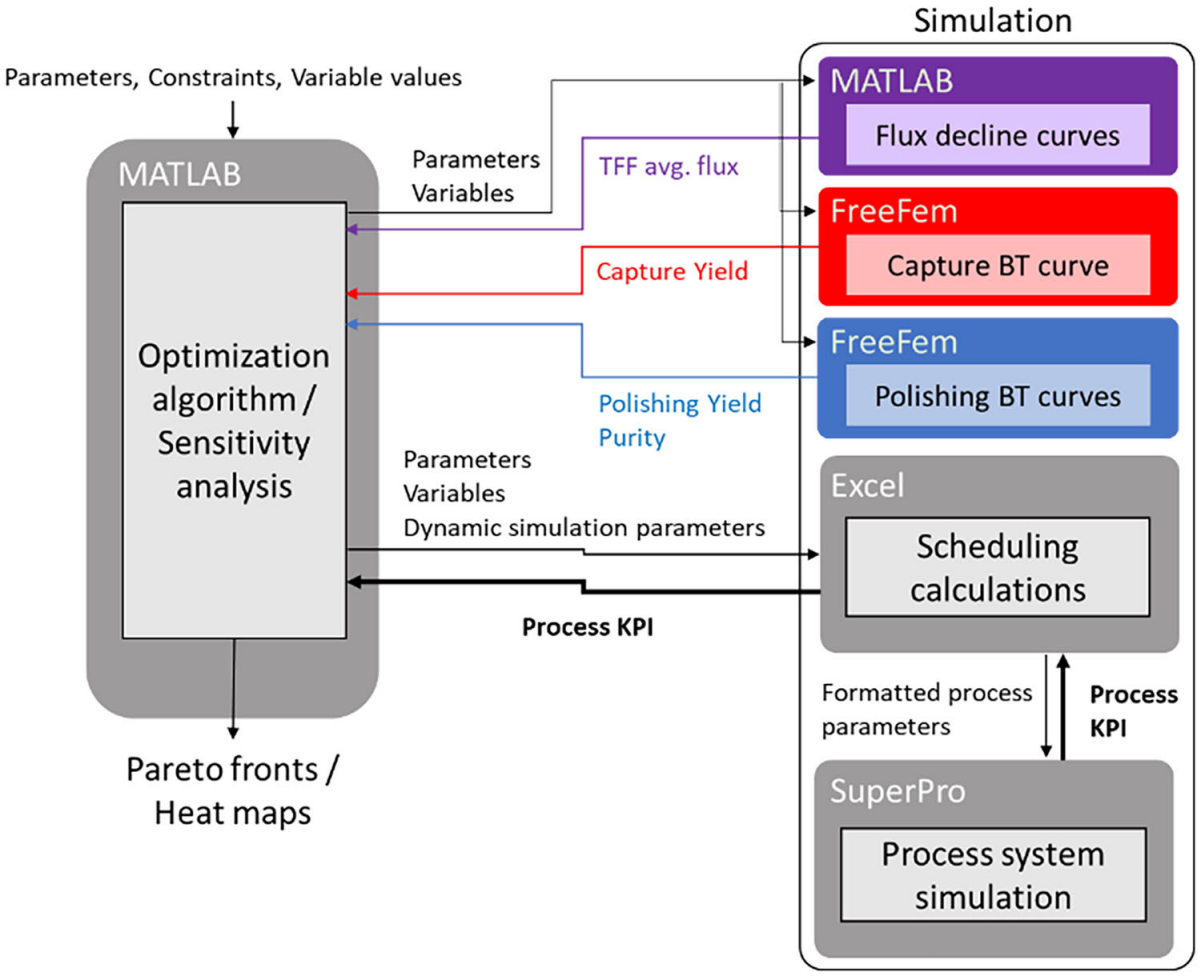
General representation of the computational framework featuring simulation blocks running in MATLAB, FreeFEM, and SuperPro Designer.

The systems of equations resulting from the previous models are solved numerically using the MATLAB ODE solver ode45 (Shampine and Reichelt 1997) for the flux decline curves and finite elements with FreeFEM (Hecht 2012) for the breakthrough curves. The parameters used are reported in Supporting Information Table S1. These solutions are used to estimate the average harvesting flux, capture step DBC, and purity and yield of the polishing step (see Supporting Information Table S2 for definitions). These parameters are then integrated into a flowsheet simulation implemented in SuperPro Designer (Intelligen Inc. 2020), which returns key performance indicators (KPI), enabling the comparison between process alternatives. Figure 1 depicts the flowchart for the framework, showing the integration of the simulation software used to perform the sensitivity analyses and optimizations. The communication between programs is set up using a shared standardized interface (COM protocol), which allows MATLAB automation to control the entire framework.

We developed a flowsheet in SuperPro (Supporting Information Figure S2) to simulate the AAV production process. The purification process includes the operations of harvesting, full and empty capsid capture, and empty‐full separation polishing. It also features two blocks to account for the non‐listed operations in the upstream process (USP) and downstream process (DSP). This simplified flowsheet lets us focus on the operations we plan to modify and compare process alternatives. The cost model parameters and main operating conditions for a base process are detailed in Supporting Information Table S3.

**Figure 2:**
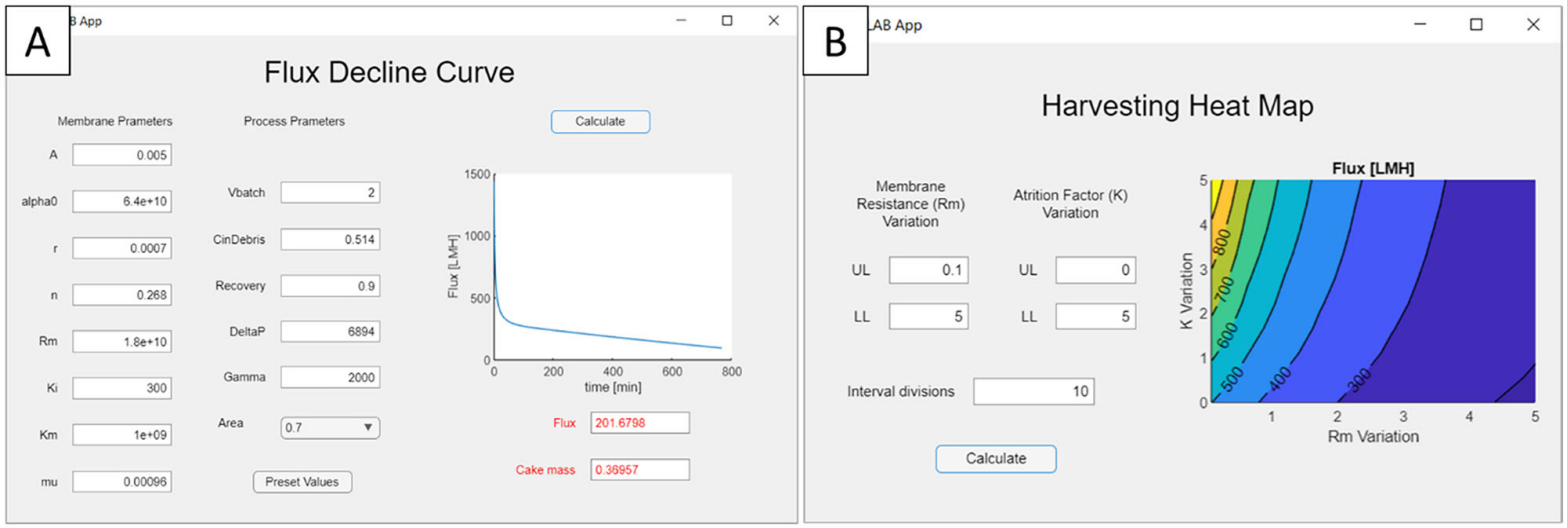
Example of the MATLAB apps developed as a GUI for the framework. (A) The GUI enables users to input model parameters and solve Equations (1), (2), (3), (4), (5), (6), (7) to simulate flux decline during harvesting. (B) Sensitivity analysis showing the impact of varying membrane resistance (R_m_) and fouling coefficient (K) on harvesting flux, visualized as a heat map.

A graphical user interface (GUI) was implemented in MATLAB to enhance framework usability. The GUI allows users to change model parameters and simulate the process to assess the impact of these modifications. For example, in Figure 2A, Equations (1), (2), (3), (4), (5), (6), (7) are solved with user‐defined model parameters, resulting in the flux decline curves for harvesting. The GUI was used to perform sensitivity analyses, where the variable ranges and number of evaluation points were specified to obtain heat maps for process metrics. For example, Figure 2B shows the impact on harvesting flux from changes in the parameters R_m_ and K (see Equations 1 and 3).

## 3 Results and Discussion

### 3.1 Harvesting

Like most operations, harvesting economics are improved by making the process faster while meeting the quality standards for the product. Harvesting process performance depends strongly on permeate flux, which impacts process speed and cost efficiency. A higher flux reduces the membrane area needed to reach a desired production rate, lowering the filtration module cost. This flux is a function of multiple variables, including operating conditions like transmembrane pressure and wall shear rate, membrane characteristics like porosity and thickness, and module parameters like total membrane area and dead volume.

**Figure 3:**
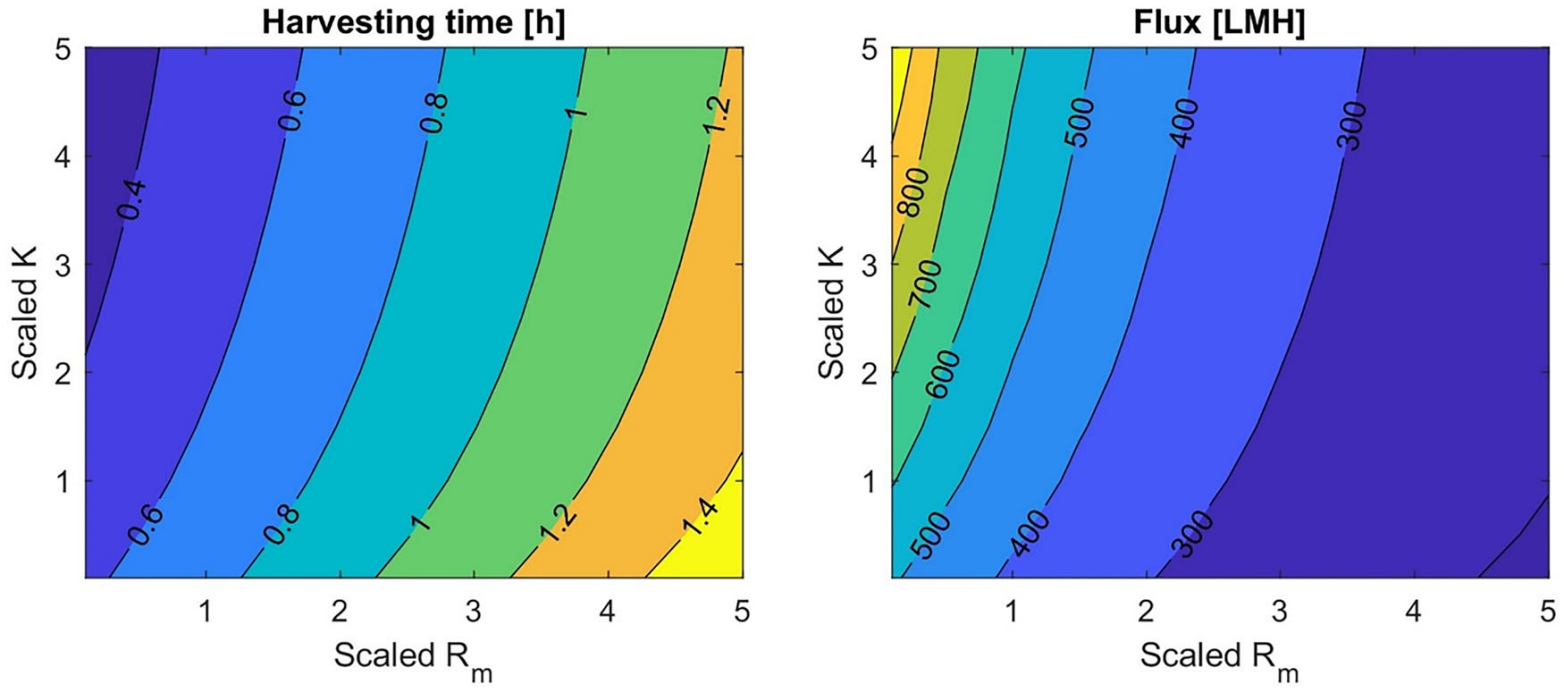
Heat maps for harvesting process time and flux (L/m^2^/h, LMH) evaluated at different values of scaled R_m_ and K. Note the significant variation in flux and inverse relationship between process time and flux.

Initially, this study explored relationships between the model parameters and permeate flux. In this context, we identified the attrition factor (K) and membrane resistance (R_m_) as important variables for module and membrane design. K offsets the cake formation during TFF, reducing resistance and increasing flux. Membranes with low R_m_ can achieve a similar result. Figure 3 shows the impact of changes in these two parameters on flux and harvesting process time. The parameters Rm, K_i_ and K_m_ are scaled by dividing by the respective reference values from Supporting Information Table S1.

As expected, the heat map shows that flux increases with lower membrane resistance and higher attrition factor. The impact on process time is inversely proportional to flux, leading to a variation of about 1 h in filtration time. This heat map showed a significant impact of the model variables on flux, with numerical results ranging from 200 to 900 L/m^2^/h (LMH) (450% variation).

**Figure 4:**
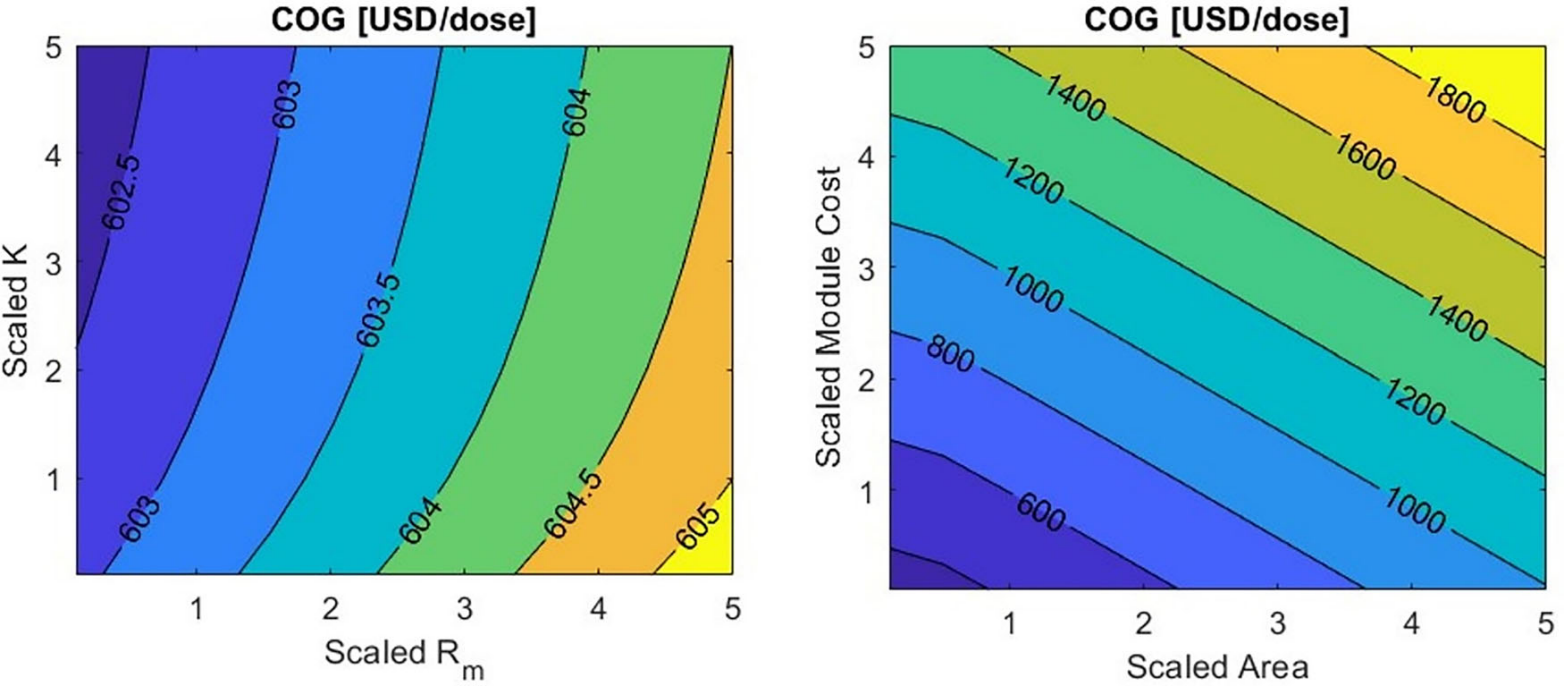
Heat maps to explore the influence of model and process parameters on COG. Note the significant difference between the two maps' scales.

Compared with other design variables, the scaled R_m_ and K variations in the range tested have a minor impact on cost of goods (COG). As shown in the heat maps presented in Figure 4, the variations in R_m_ and K yield a maximum reduction of 0.3% in COG compared with the base case. Meanwhile, variations in membrane area and module cost result in a potential COG reduction of 33%. Note that the selected ranges are arbitrary, so a 10‐fold decrease in module cost may not be feasible. Nevertheless, these results are useful for showing general trends, and the GUI allows the user to change the variable ranges according to their specific design case.

### 3.2 Capture

**Figure 5:**
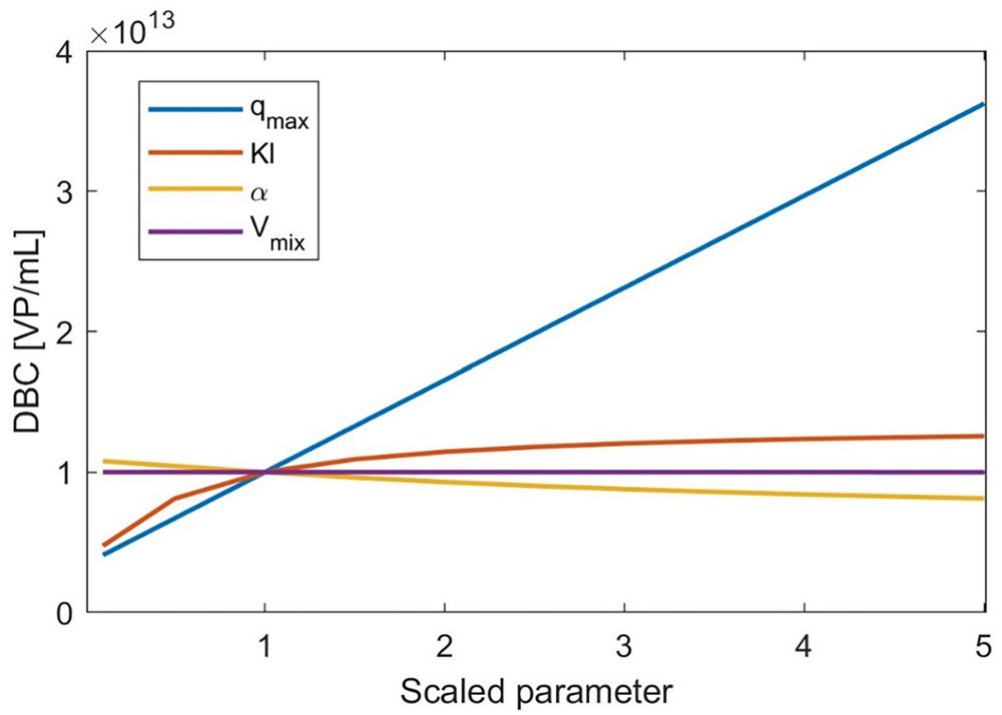
Impact of capture model parameters on DBC. The parameters are scaled by dividing by the base case values (Supporting Information Table S1). Note how q_max_ is by far the most influential parameter.

The goal in any capture operation is to separate the product from a stream containing impurities and contaminants, maximizing product recovery in the shortest time, using minimal consumables and materials. To achieve this, we seek a column with a high DBC to minimize its volume and a short RT for rapid operation. DBC (viral particles/mL membrane) is estimated using the simulated breakthrough curves generated by Equations (8), (9), (10), (11), (12), (13). In Figure 5, we see the impact of the Langmuir isotherm parameters (K_l_ and q_max_), dispersion coefficient (α), and housing void volume (V_mix_) on DBC. As expected, the maximum binding capacity (q_max_) is the most influential parameter for DBC. The equilibrium that is established between the concentrations in the liquid and solid phases, mediated by K_l_, also influences DBC, particularly for K_l_ < 1. Dispersivity has a marginal effect on DBC, as it affects the sharpness of the breakthrough curve, leading to earlier breakthroughs at high values. Finally, V_mix_ has a negligible influence on DBC, indicating that dispersion in the inlet concentration does not affect the process. Since we use an instantaneous adsorption model to describe membrane chromatography, RT does not influence DBC. This would change for resin chromatography, where rated‐based models must be used to describe adsorption.

High DBC has been the most sought‐after KPI for capture process design, particularly for mAbs, given the high cost of the chromatography media. However, economic analyses in the literature have found capture adsorber to be a minor contributor to the overall costs for AAV purification (Rieser et al. 2021; Yang et al. 2022). Despite this finding, DBC still plays an important role in process performance, depending on the approach taken for adsorber volume sizing.

In prior work with mAb process simulation, we used adsorber volume as a process variable without imposing any restrictions based on DBC (Romero et al. 2022). We expected that optimal solutions to profitability, measured by net present value, might use small columns that reduce adsorber cost at the expense of yield. For that reason, breakthrough curves allowing different yields were incorporated into the simulation. However, even with the expected lower selling prices for biosimilars, the product was too valuable to lose, and all optimal conditions displayed capture yields above 99%. The dependence of profit on yield increases with the product selling price, reinforcing that as product value rises, yield becomes increasingly prioritized over COG in all operations.

AAVs have significantly higher selling prices than mAbs, while their capture adsorber is slightly more expensive. In this context, it is crucial to have a high yield by ensuring that the feed load does not exceed the DBC. This is achieved using DBC as a constraint to define the minimum number of bind‐and‐elute cycles. In this approach, adsorber volume becomes a design variable, and COG the function to minimize, due to the competing labor costs.

**Figure 6:**
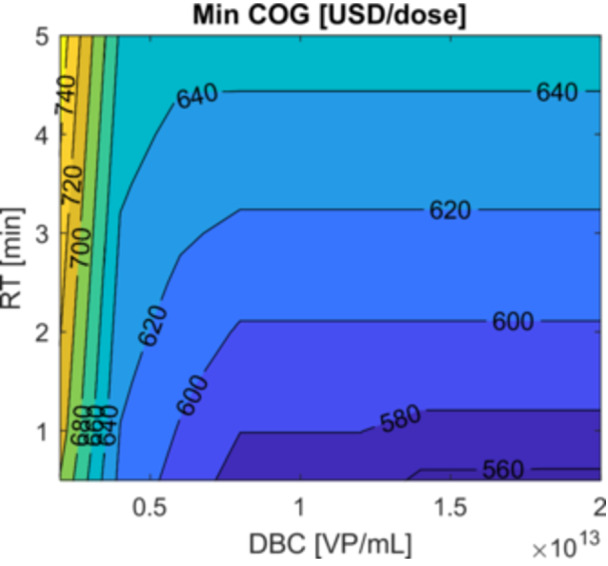
Heat map for optimal COG with adsorber volume as the design variable for different DBC and RT combinations. Note how processes featuring low DBC chromatography media can be cost‐effective if RT is low enough. However, at very low DBC values (e.g., < 0.5), further reductions in RT have a diminished impact on cost reduction.

Capture design guidelines emphasize achieving the highest DBC at the lowest RT. The key question is how much do we need to improve these metrics to observe a substantial benefit in process performance? Given the existence of optimal adsorber volumes for every combination of DBC and RT (see Supporting Information Figure S3), we must solve an optimization problem for every evaluated condition with COG as the objective function and adsorber volume as the design variable. Figure 6 shows the resulting minimum COG values. Here, at constant RT (i.e., moving horizontally), increasing DBC does not affect COG after a particular value. Initially, increasing DBC reduces adsorber volume, lowering consumable cost. However, this tendency stops when the volume reduction decreases throughput to a point where labor cost offsets any savings in consumable cost. Additionally, the heat map illustrates how, depending on the region, a low DBC can be compensated by a low RT to keep COG low.

COG is the primary KPI for capture process design, with yield and the number of doses staying constant. Regarding process time, optimal performance is achieved with the largest column and the lowest number of cycles. If volume is kept constant, we can assess the influence of RT and DBC on process time (Supporting Information Figure S4). As expected, RT is the defining variable for this KPI, as it directly governs the load rate. In contrast, DBC has a minor effect, impacting process time only through changes in the number of cycles required.

### 3.3 Polishing

Polishing follows similar tendencies as capture, with DBC and RT being important variables for process performance. Adsorber volume can be optimized based on these parameters, yielding heat maps like Figure 6. However, a primary goal of polishing is to separate full and empty capsids to increase full capsid product purity. This separation requires preferential elution of capsids from the chromatography column and collection of fractions. This fractionation may cause significant yield reduction.

**Figure 7:**
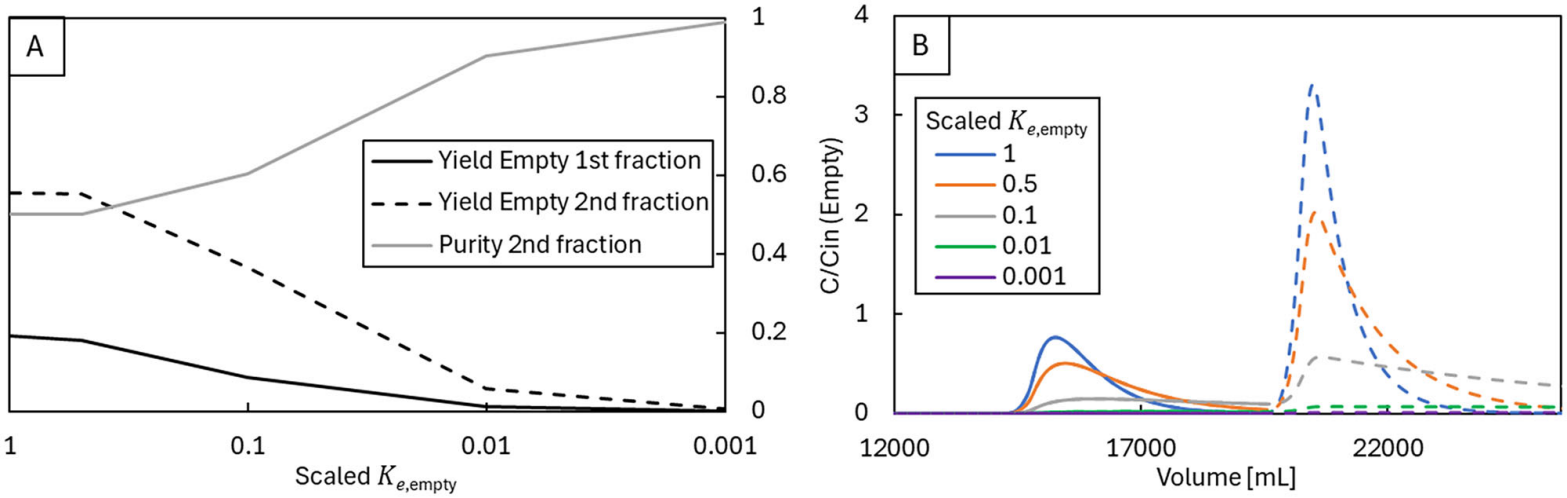
(A) Yields of empty capsids in the first and second elution peaks, along with the purity of the product fraction (second peak), as a function of *K_e,empty_*. (B) Simulated chromatograms for empty capsids with different values of *K_e,empty_*. Note the elution peaks flatten as *K_e,empty_* decreases.

We are most interested in the yield of the full capsid product, as the tradeoff between full capsid purity and yield ultimately defines the cost of improving purity. According to our model, two equations govern the elution of empty and full capsids: elution rate (Equation 14), and equilibrium solid‐phase concentration as a function of salt concentration (Equation 15). These coupled equations are independent for full and empty capsids, meaning that differences in their parameter values define their relative amount in each fraction. Figure 7A shows the empty capsids yield in the two elution fractions as a function of the elution constant for empty capsids (*K_e,empty_*), scaled by dividing by the base case parameter (Supporting Information Table S1). Figure 7B presents the simulated chromatograms for empty capsids, with variations in this parameter.

Decreasing *K_e,empty_* lowers the desorption rate of the empty capsids, causing them to elute later than the full capsids. Initially, this strategy decreased empty capsid yield in both fractions, improving purity up to 100%. In this ideal scenario, the adsorption is completely selective for empty capsids and the process behaves as a typical polishing process in flow‐through mode.

**Figure 8:**
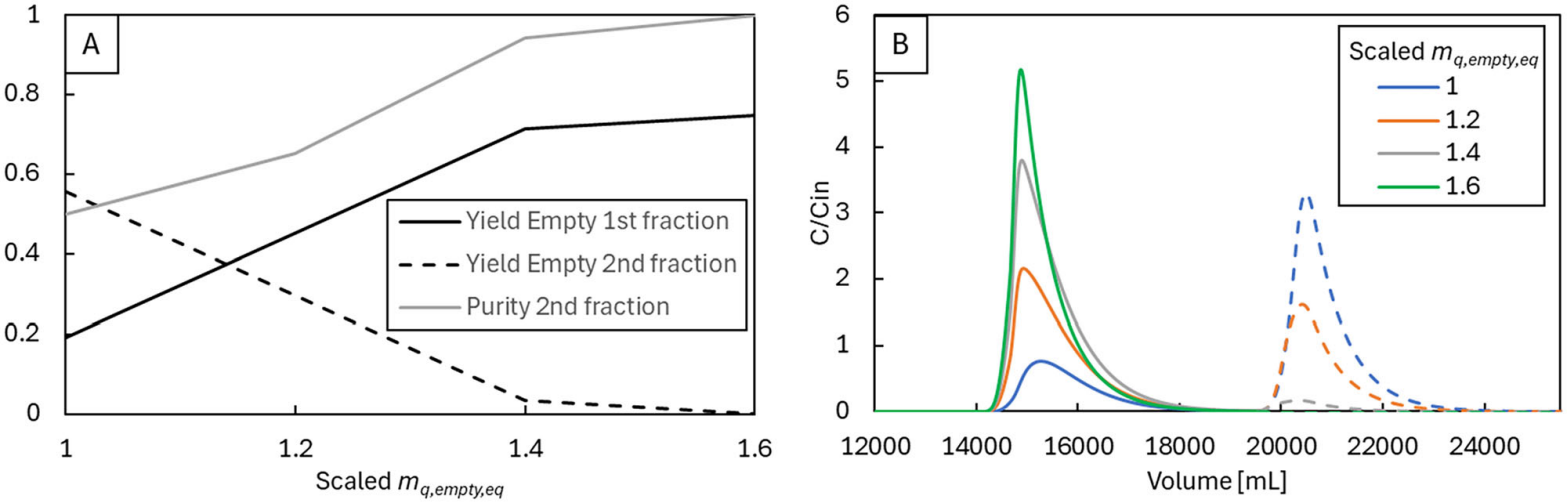
(A) Yields of empty capsids in the first and second elution peaks, along with the purity of the product fraction (second peak), as a function of *m_q,empty,eq_*. (B) Simulated chromatograms for empty capsids with different values of *m_q,empty,eq_*. Note how the model allows independent manipulation of equilibrium concentration for one species, displaying tunability not found in current adsorber options.

In practice, conductivity is changed to adjust the difference in equilibrium concentrations between full and empty capsids while keeping the elution rates constant. In Figure 8, we simulated this strategy by increasing the *m_q,empty,eq_* parameter for empty capsid equilibrium concentration. As described in Equations 14 and 15, this parameter controls the concentration of empty capsids in the solid phase for a given salt concentration. As *m_q,empty,eq_* increases, more empty capsids elute with the increase in salt concentration. If the equivalent parameter for full capsids (*m_q,full,eq_*) remains constant, this change in *m_q,empty,eq_* results in a higher empty capsid yield for the first fraction and a lower yield for the second fraction, thereby increasing full capsid product purity.

Supporting Information Figure S5 illustrates how the reduction in *m_q,empty,eq_* affects the equilibrium concentration in the solid phase and how the selected salt concentration for the first stepwise increase determines the final purity. This behavior aligns with the experimental approach that uses sequential elution to increase purity, making it a more suitable mechanism for describing the separation process (Di et al. 2024; Gomis‐Fons et al. 2024; Guapo et al. 2023; Kurth et al. 2024).

**Figure 9:**
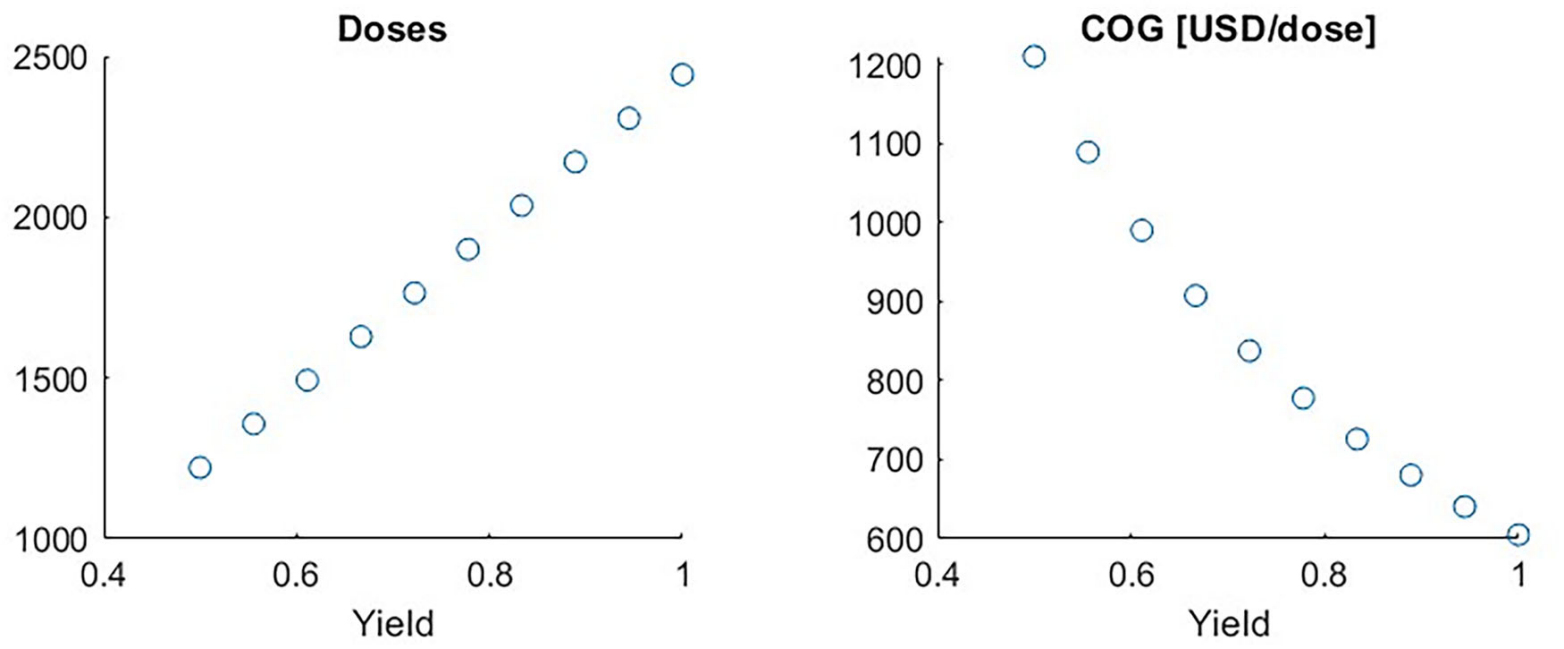
Impact of polishing yield on selected KPI. Note that this is the only sensitivity analysis that impacts the number of doses.

In the previous cases, the yield of full capsids was kept constant; however, a tradeoff between purity and yield is expected (Gomis‐Fons et al. 2024). Since purity is a quality attribute, there is no incentive to exceed the necessary level from a process performance perspective. Moreover, the FDA does not currently dictate purity due to the lack of consensus on the effects of empty capsids on AAV safety (U. S. Food and Drug Administration. 2021). Therefore, it is necessary to consider the cost associated with reducing yield to achieve a target purity. Figure 9 illustrates how yield reduction proportionally decreases the number of doses and consequently increases the COG.

## 4 Conclusions

We adapted the framework previously developed for mAb capture to simulate harvesting, capture and polishing operations for AAV vector production. While the models used can depict the general behavior of these operations, their ability to yield reliable simulations for real systems depends on estimating parameters using experimental data and validating the underlying assumptions. The GUI proved helpful in performing sensitivity analyses, enabling intuitive exploration of the design search space by adjusting variable ranges. Using a base process, we explored the impact of these design variables on number of doses and COG. This case study provided insight into the general tendencies of membrane technologies in terms of process performance, highlighting certain aspects of membrane design that could yield the most significant benefits in a production environment.

The sensitivity analysis for harvesting model parameters revealed that membrane resistance and attrition factor influence flux and, consequently, harvesting time. However, this influence did not substantially reduce total time or COG. This suggests that, during membrane/module design, focusing on high‐capacity modules (allowing higher loads per unit area) or reducing unit costs could yield greater benefits than aiming for the highest possible flux. In capture, we used DBC as a constraint, setting the minimum number of cycles needed to maintain a high yield. This strategy involved optimizing adsorber volume to achieve the lowest COG. With optimized adsorber volume, the resulting heat map for COG showed how a low DBC can be compensated by a short RT to maintain a constant COG. Finally, we compared two mechanisms for separating full and empty capsids and assessed the economic impact of increasing purity at the expense of yield loss. Although these observations are limited to a single case with specific conditions, they illustrate the effectiveness of the framework in identifying which membrane characteristics are more likely to contribute to the value proposition of new membrane technologies.

## Author Contributions

**Juan J Romero:** conceptualization (supporting), data curation (lead), formal analysis (lead), methodology (equal), software (lead), visualization (lead), writing – original draft (lead), writing – review and editing (equal). **Eleanor W. Jenkins:** conceptualization (supporting), formal analysis (supporting), methodology (equal), software (supporting), visualization (supporting), project administration (supporting), supervision (equal), writing – review and editing (equal). **Jacob I. Monroe:** conceptualization (supporting), formal analysis (supporting), methodology (equal), visualization (supporting), writing – review and editing (equal). **Ranil Wickramasinghe:** conceptualization (supporting), formal analysis (supporting), methodology (equal), visualization (supporting), writing – review and editing (equal). **Xianghong Qian:** conceptualization (supporting), formal analysis (supporting), methodology (equal), visualization (supporting), project administration (equal), funding acquisition (equal), writing – review and editing (equal). **Dibakar Bhattacharyya:** conceptualization (supporting), formal analysis (supporting), methodology (equal), visualization (supporting), project administration (equal), funding acquisition (equal), writing – review and editing (equal). **Scott M. Husson:** conceptualization (lead), formal analysis (supporting), methodology (equal), visualization (supporting), project administration (equal), supervision (equal), funding acquisition (equal), writing – review and editing (equal).

## Conflicts of Interest

Scott Husson has an ongoing financial interest in Purilogics and provides consulting services to the Company. The funders had no role in the design of the study; in the collection, analyses, or interpretation of data; in the writing of the manuscript; or in the decision to publish the results.

## Supporting information

Supplementary material ‐ clean.

## Data Availability

The data that support the findings of this study are available from the corresponding author upon reasonable request.
