# Supplementary material for: Techno‐Economic Analysis of Membrane‐Based Purification Platforms for AAV Vector Production
Source: Biotechnol Bioeng. 2025 May 29;122(9):2400–9. doi: 10.1002/bit.29034 (PMC12322620; doi:10.1002/bit.29034)
Supplement: Supplementary file 1 — Supplementary material ‐ clean. [file BIT-122-2400-s001.docx]

**Supplementary material**

**Techno-economic analysis of membrane-based purification platforms for AAV vector production**

Juan J. Romero^1^, Eleanor W. Jenkins^2^, Jacob I. Monroe^3^, Ranil Wickramasinghe^3^, Xianghong Qian^4^, Dibakar Bhattacharyya^5^, Scott M. Husson^1^

(1) Department of Chemical and Biomolecular Engineering, Clemson University, Clemson, SC 29634 USA.

(2) School of Mathematical and Statistical Sciences, Clemson University, Clemson, SC 29634 USA.

(3) Ralph E. Martin Department of Chemical Engineering, University of Arkansas, Fayetteville, AR 72701 USA.

(4) Department of Biomedical Engineering, University of Arkansas, Fayetteville, AR 72701 USA.

(5) Department of Chemical and Materials Engineering, University of Kentucky, Lexington, Kentucky 40506 USA.


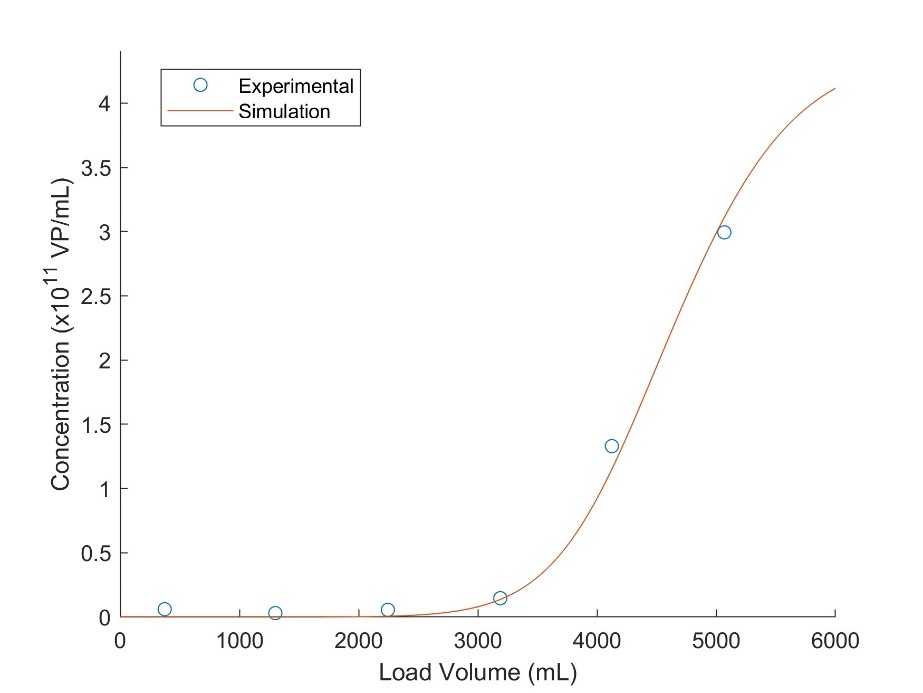


**Figure S1.** Experimental and simulated breakthrough curves for AAV capture using a Sartobind Phenyl membrane adsorber. The simulation was performed at the conditions reported in the paper by McNally et al. (McNally et al., 2020) Note how well the model follows the shape of the curve, especially at the early stages of breakthrough.


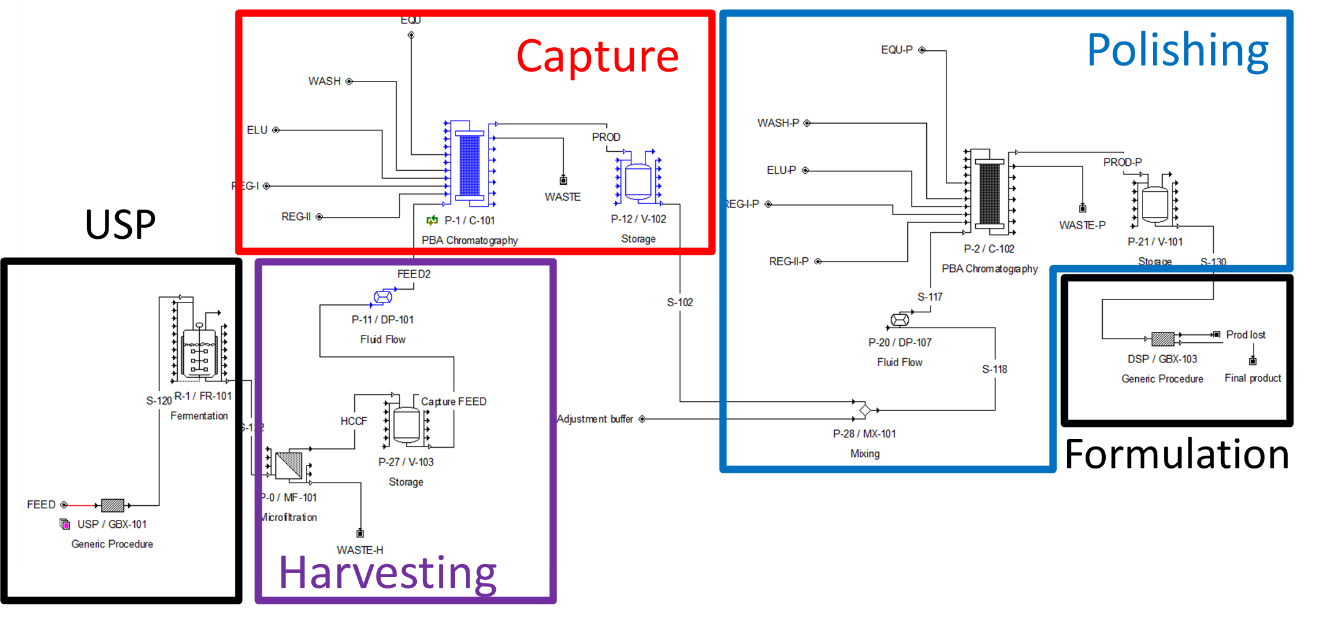


**Figure S2.** SuperPro Designer flowsheet diagram of a simplified AAV production process. Note the color correlation between the simulation blocks in **Figure 2**, which shows the origin of input information for the flowsheet simulation.


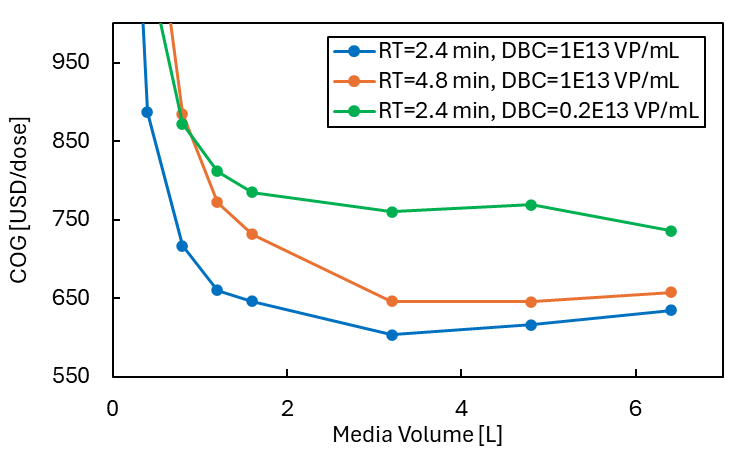


**Figure S3.** COG as a function of adsorber volume for different conditions of DBC and RT. The minimum COG for the base case (blue) is found at 3.2L, for the increased RT case (orange) at 4.8L, and for the decreased DBC case (green) at 6.4L

**Figure S4**. Heat map for capture time as a function of DBC and residence time. Column volume is kept fixed while the number of cycles are adjusted according to the DBC.


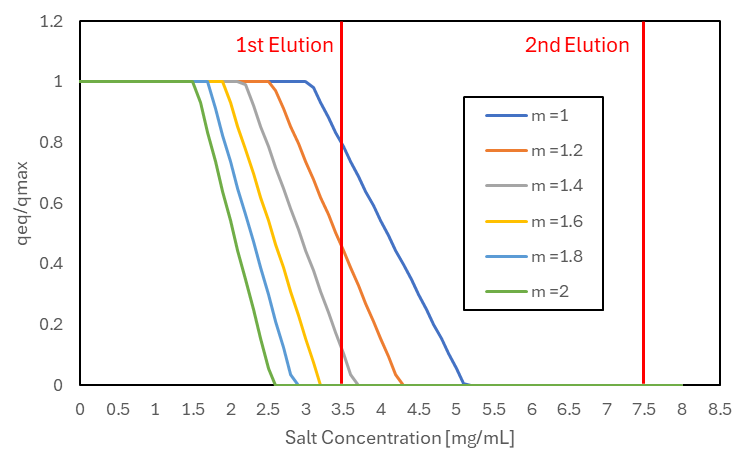


**Figure S5**. Equilibrium concentration as a function of salt concentration for different values of m_q,empty,eq_.

**Table S1** Model parameters for the base process.

| **Operation** | **Parameter** | **Units** | **Value** |
| --- | --- | --- | --- |
| Harvesting | α_0_ | kg^-1^Pa^-n^ | 6.40E+10 |
|  | n | - | 0.268 |
|  | R_m_ | m^-1^ | 1.80E+10 |
|  | K_i_ | - | 300 |
|  | K_m_ | - | 1.00E+09 |
| Capture | q_max_ | VP/mL | 1.49E+13 |
|  | K_l_ | mL/Vp | 1.83E-11 |
|  | α | cm | 0.6324 |
|  | ω | - | 0.8 |
| Polishing | q_max_ | VP/mL | 1.19E+13 |
|  | K_l_ | mL/Vp | 1.83E-11 |
|  | α | cm | 0.6324 |
|  | ω | - | 0.8 |
|  | C_s,crit,full_ | mg/mL | 3.0498 |
|  | C_s,crit,empty_ | mg/mL | 3.0498 |
|  | K_e,empty_ | min^-1^ | 7 |
|  | K_e,full_ | min^-1^ | 7 |
|  | m_q,full,eq_ | mL/mg | -0.4868 |
|  | m_q,empty,eq_ | mL/mg | -0.4868 |
|  | i_q,full,eq_ | - | 2.4892 |
|  | i_q,empty,eq_ | - | 2.4892 |

**Table S2.** Definition of process parameters obtained from mechanistic simulations.

| Operation | KPI | Definition |
| --- | --- | --- |
| Harvesting | Average permeate flux | $\frac{\int_{0}^{t} Q_{p}dt}{Filter area*filtration time}$ |
| Capture | Dynamic binding capacity (DBC) | $\frac{\int_{0}^{V} {(C}_{in}-C_{out})dV}{Column Volume}$ |
| Polishing | Yield | $\frac{Full capsids recovered in elution}{Full capsids fed}$ |
|  | Purity | $\frac{C_{full}}{C_{full}+C_{empty}}$ |

**Table S3.** Cost and operating parameters for the base process.

|  | **Parameter** | **Units** | **Value** |  |  | **Parameter** | **Units** | **Value** |
| --- | --- | --- | --- | --- | --- | --- | --- | --- |
| **General** | Capacity | Batch/yr | 45 |  | **Capture (Cont.)** | Regeneration I BV | - | 6 |
|  | Buffer cost | $/L | 3 |  |  | Regeneration I Flow velocity | cm/min | 5 |
|  | Labor FTE* cost | USD/FTE hour | 75 |  |  | Equilibration BV | - | 6 |
|  | Operators per shift | - | 6 |  |  | Equilibration Flow velocity | cm/min | 5 |
|  | Supervisory labor | % of FTE hours | 15% |  |  | Regeneration II BV | - | 9 |
|  | Lab QC | % of FTE hours | 15% |  |  | Regeneration II Flow velocity | cm/min | 5 |
|  | Utilities | USD/day | 26,100 |  |  | Membrane cost | USD/L | 19100 |
|  | Waste treatment cost | USD/L | 0.05 |  |  | Membrane replacement frequency |  | 150 |
| **USP** | Batch Volume | L | 224 |  | **Polishing** | DBC | VP/mL | 1.36E+13 |
|  | USP Cadence | days | 7 |  |  | Yield | - | 0.7 |
|  | Cin AAV | VP/mL | 1.00E+12 |  |  | Thickness | mm | 0.8 |
|  | Cin cell debris | mg/mL | 0.514 |  |  | Volume | L | 1.6 |
|  | Purity | - | 0.5 |  |  | V_mix_ | L | 2.9 |
| **Harvesting** | Recovery percentage (Filtrate/feed) | - | 90% |  |  | Load RT | min | 60 |
|  | TMP | Pa | 6894 |  |  | Washing BV | - | 10 |
|  | wall shear rate | s-1 | 2000 |  |  | Washing Flow velocity | cm/min | 3.75 |
|  | viscosity | Pa s | 9.60E-04 |  |  | 1st Elution BV | - | 4 |
|  | Lumen radius | mm | 0.7 |  |  | 1st Elution Flow velocity | cm/min | 2.5 |
|  | Membrane/module Cost | USD/m2 | 1927 |  |  | 1st salt concentration | mg/mL | 3.588 |
|  | Filter area | m2 | 0.4704 |  |  | 2nd Elution BV | - | 4 |
| **Capture** | DBC | VP/mL | 1.00E+13 |  |  | 2nd Elution Flow velocity | cm/min | 2.5 |
|  | Yield | - | 0.95 |  |  | 2nd salt concentration | mg/mL | 7.597 |
|  | Thickness | mm | 0.8 |  |  | Regeneration I BV | - | 6 |
|  | Volume | L | 1.6 |  |  | Regeneration I Flow velocity | cm/min | 5 |
|  | V_mix_ | L | 2.9 |  |  | Equilibration BV | - | 6 |
|  | Load RT | min | 2.4 |  |  | Equilibration Flow velocity | cm/min | 5 |
|  | Washing BV** | - | 10 |  |  | Regeneration II BV | - | 9 |
|  | Washing Flow velocity | cm/min | 3.75 |  |  | Regeneration II Flow velocity | cm/min | 5 |
|  | Elution BV | - | 4 |  |  | Membrane cost | USD/L | 19100 |
|  | Elution Flow velocity | cm/min | 2.5 |  |  | Membrane replacement frequency |  | 150 |

*Full-time equivalent, **Bed volumes
